# Family language policy in retrospect: Narratives of success and failure in an Indian–Iranian transnational family

**DOI:** 10.1007/s10993-023-09649-4

**Published:** 2023-02-16

**Authors:** Seyed Hadi Mirvahedi, Mona Hosseini

**Affiliations:** 1 Center for Multilingualism in Society Across the Lifespan (MultiLing), University of Oslo, Oslo, Norway; 2 Department of Foreign Languages, University of Bergen, Bergen, Norway

**Keywords:** Autoethnography, Family language policy, Heritage language, Transnational families, Translingualism

## Abstract

In this study, we investigate family language policy in a transnational family through a collaborative autoethnography. Following the theoretical underpinnings of family language policy (Spolsky in J Multiling Multicult Dev 31:3–11, 2012), we present parental language beliefs, management, and practices in retrospect to shine a light on the long-term impact of the family’s language policy on their daughter’s linguistic development in heritage languages (i.e., Persian and Hindi) and English. The components of the family language policy in this cross-cultural transnational family are sketched in the second author’s narratives of her experiences of multilingual childrearing and heritage language maintenance. We engage with, and critique, recent family language scholarship that apply postmodernist lens to examine families’ translingual use of languages at home to get by their daily life, showing how having failed to set boundaries between the home/heritage languages and English over the past nine years has resulted in their child’s predominant proficiency in English. We argue that such failure has its roots in parents’ own past lived, and future imagined, experiences, as well as language ideologies that are polycentric and scaled, the consequences of which concern emotional, linguistic, cultural and social frictions across generations. Drawing on the narratives of success and failure in the family, we call for critical adoption of translingual frameworks in examining family language policy paying careful attention to the long-term impact of such practices at home on children’s linguistic development.

## Introduction

Our contemporary world is characterized by changing political, economic and environmental conditions that bring about unequal labor opportunities and access to educational and socioeconomic capital driving families to pursue opportunities outside their home country (Hirsch & Lee, 2018). Iran is certainly no exception. Although it has been documented that some Iranians were sent to France and England over the course of 100 years (1811–1912) to study (Moradi Nejad & Pajoom Shariati, 1972), it was the upheaval of the Islamic Revolution in 1979, and later sociopolitical developments such as the Iran-Iraq war (1980–1988) and recent economic hardship imposed by political and economic sanctions that triggered emigration of Iranians in large numbers, having formed diasporic communities in different countries (Mirvahedi, 2019; Maloney, 2015). According to a report, the United States home to over 1,000,000 Iranians tops as the leading destination for Iranian migrants. Iranian migrants have also formed substantial transnational populations in Germany (around 153,000 people), England (86,000), Sweden (63,000), the Netherlands (37,500), and France (15,000) (Honari et al, 2017).

Of many key issues that families have to address in such transnational moves, the questions of what languages and how they should maintain, and start learning stand out (Duff, 2015). While heritage language(s) with their emotional value for the family will be important for family bonding and keeping in touch with the extended families across borders, as well as constructing and expressing a national/ethnic/religious identity, learning an international and societally dominant language such as English will bear implications for upward educational and socioeconomic mobility, bringing the challenging decisions about families’ language practices to the fore (Pérez Báez, 2013; Zhu & Li Wei, 2016). It is under such circumstances that family and its language policy becomes significant for developing a linguistic repertoire that will consist of at least a home/heritage language and another language of national/international value.

Despite different degrees and types of significance attached to languages as bounded, named entities by families, recent family language policy (FLP) scholarship has shifted its focus away from examining language ideologies that promote static linguistic boundaries and their impact on children’s heritage language acquisition/maintenance to how family members get by their daily life through their translingual practices. This emerging body of research examines how translingual practices, i.e. “the deployment of a speaker’s full linguistic repertoire without regard for watchful adherence to the socially and politically defined boundaries of named (and usually national and state) languages” (Otheguy et al., 2015, p. 283), could form a repertoire at the family level, *familylect*, (Van Mensel, 2018), and ultimately lead to the discursive construction of family (Hiratsuka & Pennycook, 2020). While translingual practices, along with closely related equivalents, e.g. translanguaging, is not surprising to observe in transnational multilingual families, we seek to present a case study in this paper to shed light on the long-term implications of such practices for children’s linguistic development. Drawing upon the life trajectory of a family from the Global South, an Indian-Iranian transnational family with a nine-year-old daughter, we seek to illustrate the complexities in FLPs in this transnational family, and also perhaps in other similar cases, that are informed by their own past experiences and future aspirations. Offering a thick description of decision-making processes and the concomitant practices, we would argue that translingual practices commonly observed in cross-cultural transnational families (Jenks, 2020; Lee et al., 2021) may not be adequate to build a linguistic repertoire that would consist of a well-developed heritage language (HL), and consequently English becomes the child’s dominant language in the long term. With the benefit of hindsight that the study provides, we seek to propose *family language policy in retrospect* as an insightful approach to shine a light on the long-term outcome and consequences of parents’ language ideologies, practices and management in children’s linguistic development, bridging the gap between synchronic and diachronic perspectives in FLP scholarship. *Family language policy in retrospect* allows us to critique the recent FLP studies that draw on some snapshots of translingual practices at some point in multilingual transnational families’ lives to show how family is constructed through such practices, neglecting the parents’ past lived experiences, and future imagined life, and their specific concerns and emotions, the power dimension between languages, and ultimately the long-term impact of such language practices on children’s language acquisition.

### Transnationalism, translingualism and family language policy

Studies on language practices in the family and children’s linguistic development have evolved since Ronjat’s (1913) initiation and examination of one parent, one language strategy on their own children’s bilingual language acquisition to having established a scholarly sub-field in Language Policy and Planning (LPP) in the early 2000s. As an offshoot of LPP, FLP specifically focuses on *explicit and overt planning and decision-making* with respect to language use at home and its link to child language acquisition (King et al., 2008; Luykx, 2003). Motivated by transnationalism, inevitably bringing multilingualism, globalization, and construction and expression of different identities to the fore (Duff, 2015, p. 57), FLP scholarship has burgeoned, investigating not only “explicit and overt planning” (King et al., 2008, p. 907), but also “invisible language planning” (Curdt-Christiansen, 2009, p. 352), as realized in spontaneous and fluid language planning for acquisition and use of a language (Pakir, 2003). FLP studies have shed light upon how various factors such as parents’ *language ideologies, practices and management* (Spolsky, 2004, 2012), life trajectory and future aspirations (Zhu & Li Wei, 2016), and the sociolinguistic realities outside the home (Mirvahedi, 2021a) influence family members’ agency and ultimately the bi/multilingual language acquisition of the children.

Although children’s bi/multilingual linguistic development in light of parental language ideologies and practices and home-external factors affording or constraining them has been a fundamental core element of FLP scholarship (e.g. Wright and Higgins (2022), Lanza (2007), Houwer (2007), Mirvahedi, Rajabi & Aghaei (2021), Mirvahedi, (2021b), special issues edited by Van Mensel and De Meulder (2021), Curdt-Christiansen (2013), Li Wei (2012), Lanza and Li Wei (2016), and Lanza and Curdt-Christiansen (2018)), FLP scholars in the currently emergent phase have, however, started to adopt a postmodernist lens to language planning and policy. Motivated by the colonial history of language names, this approach calls the existence of discrete languages into question (MacSwan, 2017). For this body of research, the question is not whether or not, why, and/or in what ways a child becomes bi/multilingual in light of parental language ideologies and practices, but rather, how family members draw on a “shared set of practices” containing “elements of various languages” in order to “co-create a shared family culture” (Van Mensel, 2018, p. 236). Building on Van Mensel’s notion of *multilingual familylect*, Hiratsuka and Pennycook (2020, p. 752) similarly argue that questions of heritage language maintenance or bilingual development in busy transnational families – that are “temporary assemblages of people and place” (p. 760)—are “often much less of a concern”, and such families are often concerned with “how to get multilingual family life done, and how to sustain a dynamic environment of mixed language practices rather than maintain a home language.” In light of not considering languages bounded and discrete, Wiley and García (2016) further critique parents’ attempts to raise their children through strategies such as one parent, one language policy (see also De Houwer’s (2007) critique of one parent, one language policy, which is conversely based on the premise that it does not provide enough exposure for children to learn minority languages, implying establishing stronger boundaries between languages at home).

While such depictions of flexible use of languages may unsurprisingly apply to most multilingual families, we would argue in this paper that focusing on how families talk and its contribution to daily life at home does not necessarily provide insights into the long-term impact of such practices on children’s linguistic development, parental emotions and attitudes towards their children’s proficiency, or lack of it, in certain languages and its implications for their linguistic, cultural, and ethnic identities, and family bonding (see also Jenks, 2020). FLP findings across different contexts in fact suggest that success to transmit heritage language(s) and literacy in families living away from their home country depend on the children’s constant and consistent exposure to the target language. Investigating Russian parents’ language choices and their motivation for transmitting heritage language to their children in Cyprus, Ireland, Israel and Sweden, Otwinowska et al. (2021) showed through multiple regression analyses that the transmission of Russian depended on parental efforts to actively use Russian at home and provide opportunities to communicate in the heritage language. Using a FLP framework, Dekeyser and Stevens (2019) similarly examined how the children’s family background, the parents and siblings’ language practices, attitudes, and management affect the Moroccan children’s levels of proficiency in heritage language as well as Dutch in Belgium. The analysis of the data drawn from 300 children confirmed that children’s proficiency in Dutch depended on the amount of the time they lived in Belgium, the use of Dutch among siblings, their mothers’ proficiency in Dutch, and children’s perceptions of parents’ perspectives towards the importance of learning Dutch. However, their proficiency in their heritage language was related to the parents’, particularly mothers’, proficiency and use of heritage language. Dekeyser and Stevens (2019) also suggested that children’s HL proficiency would be boosted through language management strategies such as broadening the opportunities for children to act as language brokers.

Examining a nine-year-long FLP in retrospect (diachronic perspective), and its implications for the child’s linguistic development at present (synchronic perspective), we seek to show that translingual practices in multilingual families are not special, surprising and/or unique, but their impact on the child’s linguistic development is significant. The significance of parental language ideologies and practices lie not only in their shaping the children’s proficiency in different languages, but also how they are informed by parents’ own lived experience of multilingualism, their future aspirations which interestingly reflect the power dimension between languages, and the costs (e.g. emotional) at which such goals are achieved.

### Methodology

**Figure 1:**
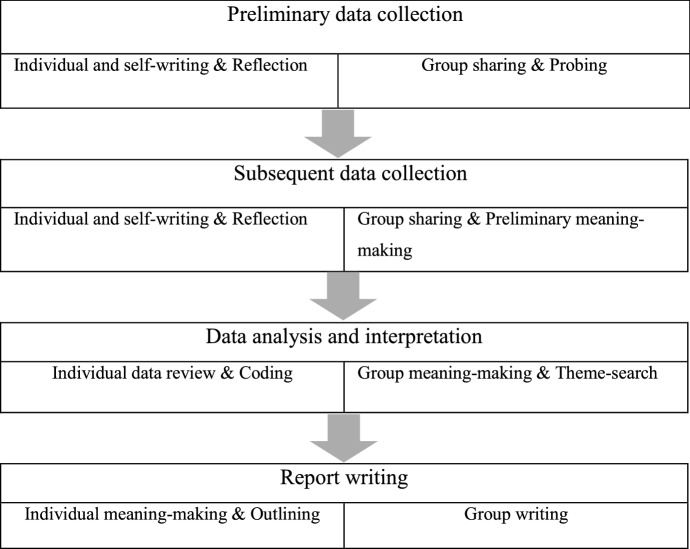
The iterative process of collaborative autoethnography (Chang et al., 2013)

This inquiry takes the form of *a collaborative autoethnography* (CAE), a form of autoethnography (AE) in which more than one autoethnographer collaborate at different stages of the study (Pheko, 2018). An autoethnography, as Ellis and Bochner (2006) explain, consists of auto (self), ethno (culture), and graphy (writing). The iterative process of collaborative autoethnography provides researchers with the opportunity to benefit from self as well as collective analysis that makes the insertion of multiple viewpoints and voices possible (Chang et al., 2013). The benefits of CAE including “collective exploration of researcher subjectivity, power-sharing among researcher-participants, efficiency and enrichment in the research process, deeper learning about self and other, and community building” help authors add rigor to their autobiographic interrogation (Chang et al., 2013, p. 25). The iterative process of collaborative autoethnography is illustrated in Figure 1.

The employment of autoethnography as a research method enabled us to narrate personal experience, focus on critical moments in our story, provide the readers with thick description, and unveil the interplay between “self” and the surrounding culture (Kubota & Miller, 2017). In the case of narrating the second author’s efforts to develop her daughter’s multilingual linguistic repertoire, including her two heritage languages, autoethnography proved to be an insightful approach since it enabled us to explore and express hidden feelings and convey them to the reader (Canagarajah, 2012).

In addition to the narrative voice, we have included two sample interactions from different phases in our story. It is through these interactions that we seek to illustrate the dynamic language practices in the family and view them in light of parental language ideologies and management. This collaborative study is a realist autoethnography in the form of layered accounts in which the fragments of experience, introspection, research, memories, and theory are put together to “reflect and refract the relationship between personal/cultural experience and interpretation/analysis” (Adams et al., 2014, p. 85). In this CAE, drawing upon a longitudinal qualitative data set collected through observations and recordings of sample familial interactions over time, we sketch a richly detailed experience of the second author’s attempts in multilingual childrearing.

In the preliminary phase of data collection, the qualitative data including audio recordings and observation notes, individually collected by the second author over the period of 9 years, were shared with the first author. The second author explained to her daughter what the research is about and sought her permission for sharing the data with the first author. Participant’s informed consent (Roulston & Choi, 2018) was thus gained prior to sharing the transcriptions, notes, and drawings with the other author. Data collection in CAE as a research method involves multiple negotiations between research colleagues (Chang et al., 2013), which was carried out through several rounds of conversations. In the subsequent stage of iterative data collection, the second author audio-recorded and observed her daughter’s language use based on the researchers’ interpretation of the data collected in the preliminary phase. The transcriptions of audio-recordings, observation notes, conversational and interactive data, self-generated personal memory data, and drawings were reflected and reviewed by the authors for the meaningful codes and themes. Prior to the writing stage, the authors created a collaborative autoethnography writing plan in which they adopted strategies about how to deal with multiple voices, ethical concerns, level of collaboration, and the responsibilities of each author (Chang et al, 2013).

In the following sections, first the family is introduced. Then, the analyses sketched in different sections are presented from the second author’s perspective in a chronological pattern according to the family’s travel history to Europe, Iran, and Malaysia. The second author portrays her experiences of multilingual childrearing and heritage language maintenance in first-person, shedding light on both diachronic and synchronic language ideologies and practices and their impact on their daughter’s linguistic development. This would form the ‘individual & self-writing and reflections’ in the process of autoethnography (see Figure 1). Towards the end of the paper, we present our collaborative ‘group meaning-making’ under the discussion section.

#### The family

I was born and grew up in Tehran, the capital city of Iran. I know Persian as my first language, and I started learning English when I was thirteen, and later became an English teacher at the age of twenty. In the second year of my teaching job, I met an Indian colleague at work, and our friendship ended up in marriage. My husband’s (Raj’s) family is from Punjab, a city in north India, but they had lived in Canada before coming to Iran. Raj himself comes from a cross-cultural family, with his mother being Iranian and his father from India. As a result, besides English, his dominant language, he can speak some Persian, Hindi, and Punjabi. He still remembers, although with a grain of bitter resentment, how his father pushed him to learn Hindi and Punjabi at home when they were in Canada, e.g. through regular phone calls to families back in India.

After we got married, rather than starting a family right away, we planned our life on studying, teaching, and visiting different countries during the New Year and summer vacations. Over the course of eight years before our daughter was born, I learned a great deal about India, and its culture, religion, and languages through our yearly visits as well as other countries, encouraging an idea of moving and living overseas, especially given our proficiency in English which would facilitate the realization of such an idea. The following sections present my narratives of the language ideologies, practices and management in our transnational family in different phases, as well as their impact on our daughter’s language acquisition and the concomitant emotions and concerns.

### Data analysis: individual & self-writing and reflections

#### *Phase 1: child’s birth and moving to Europe (Date 2012*–*2016)*

Our daughter’s (Elena) birth eight years into our marriage coupled with our plan to move overseas raised several questions. Two important interrelated issues to decide on were, first, what country we will end up living in, and second, what language(s) we will need to facilitate such a move. Our answer to the first question which was ‘one of the English-speaking countries’ such as Australia consequently informed our objective of bringing Elena up in a way that she would be equipped with the English language. However, although we both could speak English to our daughter, I decided to make efforts to raise our daughter in a way to keep her in touch with her Iranian and Indian cultures and heritages. This would require that she knew some Persian and Hindi. Yet, my husband’s past resentful experience with growing up bilingually coupled with my passion to live overseas did not afford concerted efforts to speak Persian and Hindi at home. To respect my goal of raising her with more than one language, we agreed that I would speak Persian to her. However, drawing on his own past experience, he always believed that we should never pressurize our child to learn languages. What ensued was that I resorted to various modalities of orality (Greimas, 1976), ranging from the strategy of Heritage Language (HL) input maximisation (Wilson, 2020), to taking her to Indian ceremonies and gatherings (see below).

During her first year, I used Persian, my first language, to interact with her and I read her storybooks, poems, and lullabies in Persian. As a mother, I spent more time with her since I was at home with her. At nights and during holidays, she had the opportunity to spend more time with her father. In their interactions and all activities that they did, she was exposed to English and some Hindi. As we were in Iran and surrounded by my Persian-speaking family members and friends, she also heard Persian more than other languages. This was, however, lost when she was one as we moved to Vilnius, Lithuania, where we established a business. To be with some friends, we decided to live in Frankfurt, Germany, and monitor the business in our regular trips to Vilnius. In Frankfurt, we had a couple of Iranian and Indian friends and we met them every weekend. Although Elena was inevitably exposed to some German and Lithuanian languages, Persian, Hindi, and English were the languages used at home. My main concern those days, as I recall, was that Elena did not start speaking around the time that the other children would. The only things she was able to utter were some unclear words that could make sense to me and Raj as her parents. Thus, besides reading articles and studies on children’s speech, I also sought help when we were back in Iran after three years.

#### Staying in Iran for the sake of Persian (date 2017–2019)

After almost 3 years being away from my family, we planned a short trip to Iran to visit them. Similar to most parents distressed by communication disorders who seek help from speech therapists (Skeat et al., 2010), I met with two speech therapists to understand and solve Elena’s problem while we were in Iran. They both believed that Elena’s delay in speaking was related to her confusion with the number of languages she was exposed to. Therefore, they suggested I stay in Iran for a while and only rely on Persian since it is her ‘mother-tongue’. Having bought into such myths around bilingualism (Genesee, 2015), we cancelled our return ticket and we banned using any other languages except for Persian at home. We kept trying for a couple of months, we used Persian and let her have more chance to hear and practice Persian by taking her to kindergarten for four hours every day. We noticed changes and improvement in her attempts to speak. However, we believed that her progress, rather than being related to the prohibition of English and Hindi use at home, was because of growing up, learning, and being more capable of speaking. In retrospect, this seems to have been also influenced by our future plans of living in an English-speaking country. Therefore, while we decided to resort back to the previous norm, lifting the restrictions on the use of Hindi and English at home, we enrolled her in the school of embassy of India in Iran. The decision was made initially because of its medium of instruction, i.e. English, and appreciation and supremacy they attached to it (Karimi Alavijeh & Hosseini, 2020). Apart from learning English, she would also have some exposure to the Indian community, culture, languages, songs, and dances, something that would meet my objectives. She was four and a half years old when she joined Lower Kindergarten (LKG) in the school of embassy of India. Although she kept silent at the school for the first month, she soon started speaking fluent English for her age and level. She also learned how to write in English, and within 3 months, she was ahead of all the other children in her class. I was stunned by the way she was learning and improving in English although her exposure to Persian outside the school was more than English and Hindi, suggesting the salience of the medium of instruction in language maintenance and shift studies (Mirvahedi, 2021b; Mirvahedi & Jafari, 2021). We were observing her progress in English as well as Persian and we were confident that she was on the right track for learning English and Persian.

Raj was busy with his job, and he believed that we did not need to worry and pressurize her, and she would learn everything in due course. I was more serious and determined to help her learn Persian and Hindi as her HLs and English for the future and migration purposes. Despite the language policies and practices of the school stemmed from a monolingual mindset and ‘*monolingual fallacy’* (Ndhlovu, 2015) that led the teachers to tell me to avoid speaking Persian at home since they believed it was a hindrance to English language learning, I listened to my *inner voice* to foster Elena’s HLs rather than developing only English (Curdt-Christiansen, 2014). Yet, six months into her education at the school that resulted in her increasingly proficient English, I noticed that English was infiltrating home after school hours. Initially, similar to Malay children in Singapore who use English in their imaginative plays (Mirvahedi & Cavallaro, 2020), she spoke in English to her dolls after coming back home from school, and later she extended the pattern and started using English to speak to us regardless of the languages we used to interact with her. I realized that school and the English-medium instruction had a great impact on her language choice, and how schools can touch “children’s inner world” (Vygotsky, 1967, p. 56), so I devised *pro-bilingual* plans (Altman et al., 2014) to foster Persian and Hindi. Since in the school of the embassy of India in Iran, the medium of instruction was English and Hindi was to be added to the curriculum from the third grade of elementary school, I relied on oral communication as a tool to transmit Indian and Iranian cultural practices, knowledge, religious traditions (Cohen & Twomey, 2015), as well as Persian and Hindi as her HLs.

To help her develop the Hindi language, I tried to keep connections with the Indian community in Iran which is a fairly small one constituting approximately 500 members (in Tehran), her school, and a place of worship called Gurdwara (temple of Indian Sikhs). To keep her close to the Indian culture and the Hindi language, I always took her to the religious events as well as ceremonies in Gurdwara to make her familiar with the events, ceremonies, culture, and language. Even though non-Indians were not readily allowed to participate in these events, I convinced the staff and members there to be among them. There, in Gurdwara, where she heard how they recited Indian Sikhs’ holy book and she observed their prayers, their respect to the national anthem of India, and she received warm hugs and love from the community members who called her “hamari ladki” (our daughter), she became more interested in learning Hindi. To be able to communicate with them, she asked her father to teach her more words and sentences in Hindi. Additionally, I planned activities at home with the purpose of practicing and maintaining her two HLs, which I believed, were the medium through which we conveyed our cultural values to her (Wong-Fillmore, 1991).

At this stage, as the following excerpt illustrates, our language practices at home took place fluidly, or what is often called “translanguaging” (Lee et al., 2021; Li Wei & Zhu, 2013). Yet, a closer look suggests how Elena’s attitudes towards and proficiency in English take over Persian and Hindi. The conversation took place between me, the mother (M), Elena (E), and Raj, the father (R). One evening after dinner, we started to listen to music and sing along the songs. The singing activity was Elena’s choice. We played different songs in Persian, English, and Hindi, and we sang. At the beginning of the activity, Elena warned us that she would sing only in English (line 2). Her father played the first song which was Indian, and Elena started singing along immediately (line 4). She continued with the whole song and sang along with the female singer (line 5). As the Indian song ended and her father selected the next song which was English, Elena expressed her excitement with *hooray* (line 12). She explained that she was happy because she knew English.

Persian is underlined, Hindi is in italics.

**Table:** 

1. R: OK, we will have Indian, Persian, and English song	
2. E: Man English mikhoonam haaa	2. E: I will sing English only
3. R: 3, 2,1, just sing whatever is played	
4. E: *Usne Mujhe Chhua Bhi Nahi* [starts singing the song]	
5. E: I don’t sing the part that the man sings, dad you sing those parts	
6. R: Ok, I will sing this part	
7. M: Pas manam migam 1 2 3 [The repeated part of the song]	7.M: I will just count 1, 2, 3 [the counting is repeated in the song]
8. E: Ba’azi jahash stop mikonam chon nemitoonam bekhoonam	8.E: I stop in some parts because I can’t sing
9. R: Ok, no problem, whatever you know	
11. R: Now, let’s go to English song	
12. E: hooray, I know English	
13. R: Believer	
14. E: YES [you could see the sparkles in her eyes]	
15. M: Role(e) man chie baraye believer?	15.M: What is my role for believer?
16. R: you can play the drums	
17. M: ok	
18. E: First things first I'mma say all the words inside my head	
19. M: Ok bacheha, berim baraye Farsi?	19.M: Ok guys let’s sing Persian
20. M: [after 2 min], Bacheha berim Farsi?	20.M: Guys! Let’s start Persian song
21. R: Which Persian song?	
22. E: Farsi baraye to maman, man nemitoonam bekhoonam	22.E: Mom, Persian is for you, I can’t sing
23. R: Ok, copy, just repeat whatever you hear	
24. M: Yani hichi ahange Farsi balad nisti?	24.M: You mean you don’t know any Persian?
25. M: Not even one song?	
26. E: You sing, you know Persian better than me…	
27. M: Ok, we can replace Persian song with another Indian song that we both like “Ashiqi”	
28. E: So, Persian is cancelled!	

The song started and Elena sang excitedly with a louder voice than the Indian song, she did not even take a breath, meanwhile, she signaled me to play the drums. It seemed a great deal of her happiness and excitement were related to her competence in English compared to Hindi. One potential reason could be that English was the medium of instruction and literacy at school. Her great enthusiasm declined as the song finished and she realized that the next song would be a Persian one. As she was singing the last verse of the song, I told her that the next song will be Persian, however, she kept singing “Believer” without any reactions to what I said. She lifted her eyebrows which meant a big no to her father’s suggestion of Persian song. Although her father noticed Elena’s unwillingness for the Persian song, he insisted on playing. As Elena found out that her father was determined in playing the Persian song, she expressed her dislike for Persian to me and asked me to take her role of singing (line 22). I could see how desperate she felt when she was pushed to sing in Persian. All the happy moments that she was experiencing during family activity vanished. She was just trying to convince her father that she would not be able to sing in Persian. Although I planned the activity for her to have a practice of Persian and Hindi, I did not want to see her in despair. Therefore, I suggested skipping the Persian song (line 27). The fluid nature of language decisions in our family did not contain any explicit and overt planning, rather it was a dynamic and emerging product of the roles that we took while parenting (Higgins, 2018) in which both of us as parents sought ways to maintain our HLs and keep Elena connected to the languages as well as the cultural backgrounds. However, in some cases, my language belief was not reflected in my language practices (Piller & Gerber, 2021), particularly when I felt multilingualism was a source of anxiety (Sevinç & Mirvahedi, 2022; Sevinç, 2022), I doubted our FLP, and to lessen the anxiety, I reduced Persian language input and expected less output. The language practices were thus transformed into English, either by me switching to English (lines 25 & 27), or engaging in a “dual-language interaction” (Nakamura, 2017) where I used Persian to address her or Raj but they responded in English (lines 15–19).

The sample conversation illustrates how her 18-month schooling and becoming increasingly more proficient in English enabled her to resist our language planning (Smith-Christmas, 2020a). Her agency in shaping language practices often took the form of me engaging in ‘adult code-switching’ (Lanza, 2007), which was triggered by witnessing her anxiety in understanding my words in Persian. Language practices at home were even more skewed towards English when we moved to Malaysia.

#### Phase 2: moving to Malaysia (dates 2019–2021)

Elena studied in the school of embassy of India for almost 2 years and when she became 6, we moved to Malaysia, where I believed the presence of Indians as one-third of the population would still support her ties with Indian culture and languages. Following the salience of English for our goals, we enrolled Elena at an international primary school where the medium of instruction was English and French was taught as a second language. Having been exposed to English at school for 8 hours and cut off from her Persian-speaking environment, she became increasingly more reluctant to use Persian at home. Just about 3 months after our residence in Malaysia, I noticed that she evaded the frequent video calls I made with my family members. In response to my requests for communicating with them, she explained how anxious and frustrated she felt when she was not able to talk to them as fluently as she did when we were in Iran. In contrast to studies showing the positive impact of video calls in heritage language maintenance (Palviainen, 2020; Said, 2021), video calls did not play an effective role in maintaining Persian in our family. Six months into our stay in Malaysia, when she was explaining what happened to her at school, she began struggling with Persian words, telling me “mum, I don’t think I can speak Persian anymore, let me explain in English.” There, I understood how her 8-hour daily interactions in English at school was causing her ability to speak Persian to diminish day by day as it was a non-societal language in Malaysia. Although I felt a pain in my heart about what was happening, I pretended that I was quite satisfied if she preferred to explain in English. My concern, rather than the disappearance of Persian from her language repertoire, was tied with how I would be able to keep her in touch with Persian literature, poems and poetry, and the culture which are interwoven to language.

To make our dream of living in an English-speaking country come true, Raj applied for a position in Australia and we planned to move. After we were granted the visa, we decided to move to Iran and wait for the Australian Covid-19 border restrictions to be lifted. I remember how worried and anxious she felt when she was not fluent in Persian, but her cousins who knew English in my family assured her that they would understand her despite the constant code-switching in her speech. Elena did not have any sense of belongings to Iran, her homeland, because of our family’s mobility and temporary stays in different countries. Therefore, anxiety, shyness, and psychological challenges which are related to the use of heritage language among members of an immigrant community (Sevinç, 2018) were obviously observed when she had to use Persian. However, she made a great progress in Persian within the first 2 months of arrival, suggesting that she had some level of “hidden bilingualism” (Nakamura, 2016) that could be activated when the need arose.

As the border restrictions extended, we decided to enrol her at school in Iran. With no literacy in Persian, she could not study in schools in Iran. Besides, we were supposed to be in Australia within some months. She could always join the school of embassy of India again; however, in search of a quality school, I found out that an international school has been established in Tehran. The school catered students from families with, at least, one of the parents holding a passport from a country other than Iran. The school was doing a professional job in terms of recruiting qualified teachers, providing English materials to teach, and developing a curriculum which was obviously different from the one in Iranian schools. Elena studied there for five months, after which we moved to Australia where she joined a public school. It was around this age that I could observe not only did her English proficiency dominate Persian and Hindi, but she had also acculturated to non-Iranian customs and traditions. The following excerpt illustrates the point. Note that at this point, I had also stopped speaking Persian to her.

#### Acculturation to non-Iranian customs, traditions, and values

**Fig. 2:**
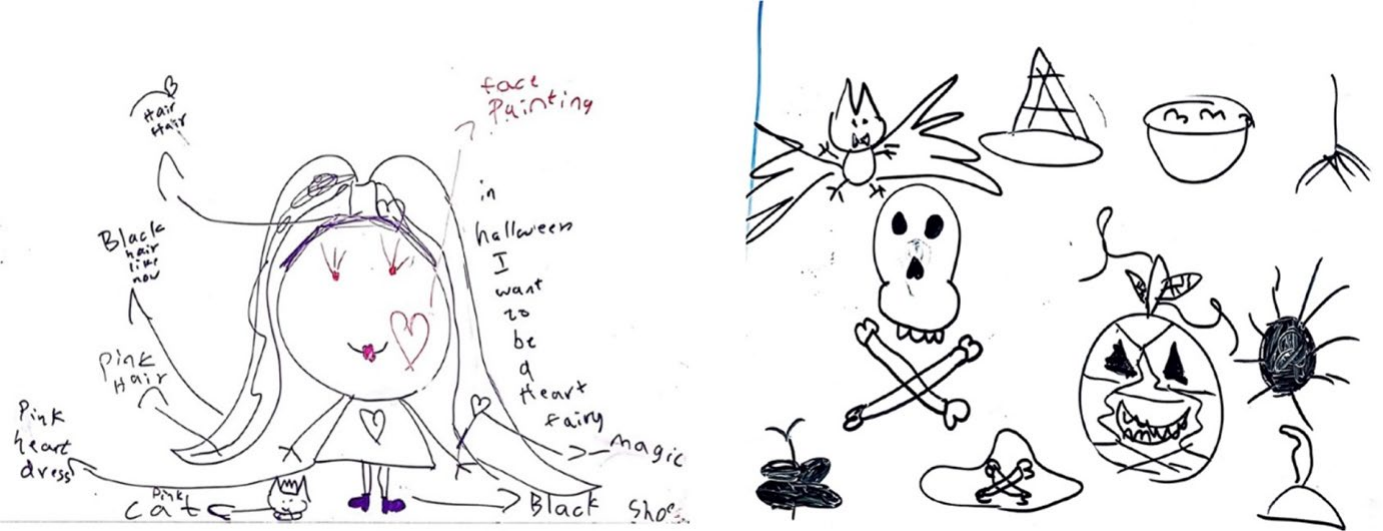
Elena’s plan for Halloween

It was a hot, sunny day in August 2021, the time when Elena was 9. I entered her room and I noticed drawings on her whiteboard (Figure 2), and when I asked her about her drawings on the board, she explained to me that she was planning her dress for the next Halloween. I reminded her that we might still be in Iran due to Covid-19 border restrictions in Australia and she might have to use last year’s stuff used in Malaysia since Halloween stuff are not readily available in Iran. As our conversation continued and she expressed her sadness of being in Iran as well as her desire to leave Iran before Halloween, I tried to remind her of some other cultural events in Iran and India such as Norouz (the new year in Iran) and Deepavali (the festival of lights in India).

1. M- Near Halloween, there is another event, do you remember?
2. E- No
3. M- We used to attend it in Gurdwara (in Iran) and Mid Valley Mega mall (in Malaysia) to see the event and the decoration
4. E- Aha … Deepavali … the Indian event
5. M- Are you more excited about Deepavali or Halloween?
6. E- Well, I mostly like Halloween and Christmas
7. M- Aha, why?
8. E- Because we wear red, white clothes, and have Christmas trees
9. M- What about Norouz (Eid) in Iran?
10. E- I prefer Christmas. Norouz is also good but mostly I love Christmas
11. M- Why do you love Christmas but not Norouz?
12. E- Because we do not have Christmas trees, no colorful dress
13. M- Do you have special memory about Christmas that made you love it?
14. E- YES [VERY EXCITED], When we were in Malaysia, at school I sang song in Christmas, so I loved it
15. E- In Norouz, we didn’t do anything here… we just visited the family and received money in envelope …it’s boring I don’t like I mean I like but I prefer Christmas
16. E -What about Deepavali?
17. E -Hmmm… [She seems to be hesitant and she has mixed feelings. Her facial expression and her silence make me dig more and ask more questions]
18. M—Don’t you like it?
19. E—In Malaysia? NO, I didn’t like it!
20. M -Why? When you were in Iran, and you joined Kendriya Vidyyalaya (KV) you loved Indian celebrations. Why didn’t you like it in Malaysia?
21. E -You know in KV in Iran, I was the first and top of everyone… so, they preferred me and gave me awards and I liked it in Iran more.
22. E—But in Malaysia, there was singing and dancing, they didn’t give me singing and dancing activity, they gave one of my Indian friends! I’m also a good dancer and can sing Indian songs!
23. M—What if you go to another school and there, they let you participate in Indian activities?
24. E—Yes, then I will like it again, but if others dance, I won’t like it

As I was trying to remind her of the ceremonies and cultural values in Iran and India, she was convincing me that both Norouz and Deepavali are not among her favorite events compared to Christmas. I tried to remind her of our memories of these events and she was explaining the history and beliefs associated with Halloween in response. In our dialogue, I noticed how she was acting as a powerful social actor and *language and culture broker* at home (Orellana, 2009). However, she was doing it cautiously not to hurt my feelings and she had different reasons for each of her dislikes. While she found Norouz boring and colorless compared to Christmas when we can decorate trees and wear red dresses (lines 8–15), her negative attitude towards Deepavali was shaped by a memory in Malaysia (lines 22–24). I assume that her agentive use of the English language, the expression of her enthusiasm for Halloween and Christmas, and her unwillingness to speak Persian and engage in Iranian traditions and ceremonies were associated with *linguistic norms* of the educational settings where she had been schooled (see also Mirvahedi, 2021b).

More surprisingly, in my conversations with her about the drawings which indicated her plans for the upcoming Halloween, I also realized that joining the school in Malaysia influenced her attachment to the Hindi language and her identity. For the first time, she articulated how she tried to convince teachers at school that she was Indian and she would like to be in the Indian dance group (16–24). The teachers, however, selected her for Ribbon dance which was not of her interest. I was wondering how the teachers judged her and placed her in another dance group although she explained to them that she held an Indian last name and her father is from India. I just could think of two reasons; her appearance which resembles me and my interactions with teachers who knew me as an Iranian. Previously, she was in the Indian music and dance group in the school of the embassy of India in Iran and she also won the award for best singer and dancer from Kindrya Vadialia (KV) central office in Hyderabad when she was 6. In Malaysia, however, everything changed. The majority of the Indians in Malaysia were from South India and had a different language, religion, complexion, and culture from people in North India where Elena’s father is originally from. Therefore, at school, they did not consider her to be an Indian student. Her involvement in activities such as the dance for Christmas and Halloween within a 2-year-period and ignoring her Indian identity made her more detached from the Hindi language and Indian culture. One could argue here that Elena’s detachment from the Indian culture and Hindi language resulted from the impact of macro-societal ideologies and institutional policies on her identity (Baxter, 2016). What we were trying to build for the past six years was almost shattered during our two-year stay in Malaysia. It was then that we realized how the macro-level societal ideologies overshadowed our family language practices and management at the micro-level (King & Fogle, 2013). These thick descriptions of my experiences of multilingual childrearing and heritage language maintenance in this section paved the way for our collaborative, group meaning-making in autoethnography. At this stage, we shared and reflected on my individual sense-making of the data including transcriptions of audio-recordings, observation notes, conversational and interactive data, as well as *self-generated personal memory data.* We reached agreement on our interpretations of the layered accounts which we now present as discussion and concluding remarks in the following sections.

## Discussion: group meaning-making

Our discussion of the lived experience of linguistic repertoire is built on three important questions raised by Busch (2017): (a) How is an individual’s linguistic repertoire constituted, and what linguistic baggage they bring as speakers when they move across spaces? (b) How do discourses about language and ways of speaking as well as ideologies that shape the space position the speakers, and how do the speakers position themselves in regard to these discourses and ideologies? And c) With what feelings and bodily sensations do the speakers experience themselves as speakers? We have argued in this paper that FLP scholarship examining translingual practices at home without considering their long-term impact on children’s acquisition of languages and the emotions and concerns around it inevitably focuses on the first question, investigating how linguistic repertoire at the family level emerges and how this repertoire discursively constructs the family. They thus fail to acknowledge and systematically analyze how discourses about language and linguistic ideologies in the family and beyond – which can reflect the power dimension between languages and speakers—have a significant impact on the children’s linguistic development, and how parents and children live the languages they know and learn through emotionally-loaded experiences. This, as Jenks (2020) argues, also highlights conceptual and practical gaps between poststructuralist and postmodern constructs on the one hand, and ideological positions based on individual life trajectories, on the other, begging for further research to offer practical solutions, guidelines, or advice for families.

Drawing on the thick descriptions of language ideologies, practices and management and the concomitant challenges, concerns, and outcome in different phases of the transsettler family, that is, “transnationals whose moves are initially not viewed as necessarily permanent” (Hirsch & Lee, 2018, p. 3), we have attempted to contribute to addressing these theoretical questions and gaps. Auto-ethnographic longitudinal qualitative studies lend themselves very well to this line of inquiry as they connect personal lived experiences with the wider cultural, political, and social meanings (Liu & Li, 2019). Our study has revealed how language policies at home, though being ultimately “self-induced and self-policed modes of ‘order’ in social action,” are infused by “polycentric and scaled language ideologies, accumulated and learned during biographically phased processes of socialization” (Blommaert, 2019, p. 5). In particular, considering the parents’ own lived experiences that inform their language ideologies is of paramount significance in studies that involve families from the Global South (Lomeu Gomes & Lanza, 2023) whose mobility would depend to a great extent on their proficiency in an international language such as English. As we observed, the father who himself grew up in Canada as a migrant in a multilingual family had a resentful experience of being pushed to develop language skills in heritage languages (Hindi and Persian) and English. His negative memories of being expected to speak heritage languages at home shaped his ideology of not ‘pressurizing the child’ to speak heritage languages at home. By contrast, while the mother’s monolingual upbringing in Persian gave her stronger attachment to the Persian language and culture, making her seek to maintain it, her interest in English developed in her adolescence in English classes and later her view of it as a language that could secure future socioeconomic mobility made her invest in her child’s English since her young age. Moreover, both parents’ proficiency in English and Persian, though to different degrees, facilitated their marriage in the first place that brought about a new *biographically phased processes of socialization* during which new communication norms came into existence, shedding light on the salience of family configurations in shaping language ideologies and practices at home (Altinkamis, 2022; Vorobeva, 2021). In other words, if both parents were from the same language background and experienced similar processes of socialization, the odds of more exposure to the HL and hence its acquisition by the child would have increased. In this family, although the father had learned some Persian from his mother and improved after his move to Iran and marriage to his Persian-speaking spouse, that does not seem to have contributed significantly to their daughter’s acquisition of Persian.

The long-term impact of language practices on children’s linguistic development has been also shown to be inextricably linked to emotions, an important dimension of FLP that is neglected in ‘peaceful’ description of multilingual families’ language use, who, in Wiley and García’s (2016, p. 59) terms, “simply translanguage”. Rather, parents have always certain emotions and concerns about their children’s linguistic development, both about the process and outcome of such development (Sevinç, 2018, 2022). Our findings show that, for example, the mother was concerned when their daughter’s speech was delayed, and at a later stage, she experienced a pinch of regret that her daughter increasingly loses her linguistic capacity to stay in touch with her grandparents in Iran. The narratives show that Elena herself also experiences anxiety and despair when it comes to communicating with Persian speakers in Iran. Ability to speak a heritage language is not, however, only about communication. A heritage language can be drawn upon by a person, or family collectively, to identify with a certain culture, religion, or ethnicity with their own specific traditions, values, and practices (Little, 2020). The friction across generations could occur exactly when they do not share the same linguistic repertoire to create such identities and engage in cultural performances. Gharibi and Mirvahedi (2021), for example, show how some children of Iranian families in the UK consider themselves to be British, while the mothers worry about ‘losing them’ as they increasingly engage in a British way of life. Similarly, Elena’s increasingly dominant proficiency in English has shifted her preferences for, and attitudes towards different cultural values, making her like Christmas over Nowruz or Deepavali. Accordingly, unlike to findings suggested by Et-Bozkurt and Yağmur (2022), affordances such as the Internet and social media making it possible to stay in touch with families and relatives could not compete with the significant impact of the educational policies favoring English over other languages in the international schools.

## Concluding remarks

Nine years into Elena’s journey of linguistic development, we can observe that the absence of a strong pro-heritage language policy to prioritize the language at home and also strategies to utilize home-external affordances, such as schools, in favor of the heritage language, has not resulted in an active bilingualism in English and HLs. In line with De Houwer and Bornstein (2016) and De Houwer (2007), our observations also show that regular, constant and meaningful input and interaction as well as established pro-HL parental discourse strategies (Lanza, 1998) are needed so that a child can learn and use a language. This is in particular of paramount significance in high-stake settings when a language is marginalized, such as a heritage language in a highly mobile family. In this case, having boundaries between languages in the family, especially in early childhood, actually helps establishing bi/multilingualism in the long run. In other words, future bi/multilingualism and capacity to translanguage later in life will be made possible by viewing languages as discrete and bounded in the family, and engage in monolingual practices to promote the marginalized/minority language in the child’s early years of linguistic development.

Finally, while the attempts to promote social justice in pedagogy through translanguaging cannot be neglected, we have argued that adopting translanguaging and similar other trans-constructs in the family should be done with care and critically, considering their linguistic, emotional, political and idenity-related long-term impact for both the children and the parents in the families who seek and hope for their children to develop a bi/multilingual repertoire consisting of their home/heritage language(s). Multilingual families certainly draw on their linguistic repertoire to carry out their daily life; nontheless, as we have shown, if boundaries are not set for a marginalized/minority language (Bonnin & Unamuno, 2021), the child may not develop a linguistic repertoire consisting of that language, bringing about different emotinal, linguistic, cultural and identiy-related frictions in the long run. Robust longitudinal research including FLP studies in retrospect deem necessary to understand to what extent viewing languages with no boundary between them and translingual practices in multilingual families can lead to richer linguistic repertoire, one that will have a home/heritage language so that the objective of fostering social justice through translanguaging for marginalized languages can be established at home as well.
